# Morphological, physio-biochemical, and molecular indications of heat stress tolerance in cucumber

**DOI:** 10.1038/s41598-023-45163-7

**Published:** 2023-10-31

**Authors:** Eman El-Remaly

**Affiliations:** Cross-Pollinated Vegetables Research Department, Horticultural Research Institute, Agricultural Research Center, Giza, 12619 Egypt

**Keywords:** Genetics, Plant sciences, Climate sciences

## Abstract

Global warming is a critical challenge limiting crop productivity. Heat stress during cucumber growing stages caused deterioration impacts on the flowering, fruit, and yield stages. In this study, “inbred line 1 and hybrid P1 × P2” (heat-tolerant) and “Barracuda” (heat-sensitive) were utilized to determine the heat tolerance in summer season. The heat injury index was used to exhibit the heat tolerance performance. The heat injury index for heat tolerant (HT) genotypes, on leaves (HIIL%) and female flowers (HIIF%), was less than 25 and 15 % in HT, compared to heat sensitive (HS) was more than 75 and 85%, respectively. Moreover, the content of leaf chlorophyll, proline, brassinosteroid (BRs), abscisic acid content (ABA), the activity of catalase (CAT, EC 1.11. 1.6), peroxidase (POD, EC 1.11.1.7) and superoxide dismutase (SOD, EC 1.15.1.1) increased with the heat stress responses in HT plants. Expression pattern analyses of eight genes, related to POD (CSGY4G005180 and CSGY6G015230), SOD (CSGY4G010750 and CSGY1G026400), CAT (CsGy4G025230 and CsGy4G025240), and BR (CsGy6G029150 and CsGy6G004930) showed a significant increase in HT higher than in HS plants. This study furnishes valuable markers for heat tolerance genotypes breeding in cucumber and provides a basis for understanding heat-tolerance mechanisms.

## Introduction

The world faces an extraordinary challenge in the aspect of climate change, including increasing temperatures. Global warming warns of a serious deficiency in the production of crops, particularly food^1^. It is assessed that a 1 °C raise in seasonal temperature can directly refer to the loss of 2.5–16% of main crops in tropical and subtropical regions^2^. Heat stress has destructive impacts on plant growth and productivity^3^. Therefore, efforts must be made to address this challenge and find alternatives that mitigate these expected losses. The efforts of plant breeders come in a progressive rank in developing high-quality varieties that are tolerant of harsh changes in the environment and capable of continuity and sustainability of production^4^. Cucumber is one of the numerous sensitive heat crops. The high temperature is one of the most destructive, adverse conditions for cucumber production in open fields and greenhouses^5^. In summer, the temperature in open fields naturally exceeds 38 °C, and in the greenhouse surpasses 45 °C which shows to leaves sunburn, growth retardation of stems and roots, fruit miscreation, and plant death, which harshly affects cucumber yield and fruit quality^5,6^. Heat tolerance mechanisms in cucumber need deep explanations to debate the defense approach. Little progress has been achieved in cucumber heat tolerance mechanisms explanation, on adult cucumber plants to spot gene expression associated with extreme temperature stress^7,8^. The heat injury index was documented as the indicator of heat tolerance of cucumber at the seedling stage^9^. Chlorophyll is susceptible to many abiotic stresses, including temperature and humidity^10^. Plant hormones play a critical role in heat reactions, Abscisic acid (ABA) is a phytohormone that relieves the adverse impacts of heat stress by reducing oxidative injury and supporting photosynthesis^11^. Brassinosteroids (BRs) are a group of plant steroids that are imperative for a broad range of cellular and physiological processes, including stem extension, pollen tube growth, leaf bending, root inhibition, fruit growth, ethylene biosynthesis, proton pump activity, xylem differentiation, photosynthesis, gene expression, and mitigate heat stress^12^. The biochemical and physiological consequences of heat stress include the extra accumulation of reactive oxygen species (ROS)^13^. The oxidative damage and irreversible denaturation of proteins result in protein misfolding, aggregation, and alterations to the lipid membrane leading to damage in membrane permeability and raft disruption^14^. Undoubtedly, the progress of plant heat stress tolerance could be connected to increasing antioxidant enzymes^15^. In order to annihilate the ROS damage, plants have developed the antioxidant enzymes that are responsible for scavenging superfluous ROS accumulation under environmental pressures, including, Catalase (CAT), superoxide dismutase (SOD), and peroxidase (POD)^15^. Heat stress motivate the production of proline, which acts as an antioxidant^16^. The chlorophyll metabolism and protection reactions were up-regulated in thermos-tolerant plants, while they down-regulated in thermos-sensitive plants^17^. Gene expression investigations for genes that are responsible for enzymes and plant hormone secretion under heat-stress conditions are excessively higher in heat-tolerant plants than in heat-sensitive plants at all growth stages in cucumber^18,19^. The family genes of Dof zinc finger protein DOF5.7 have confirmed roles in heat stress tolerance^20^. These families have been indicated to play vital roles in numerous biological operations, such as the synthesis of seed-hold protein, seed development, germination, and flowering^21^. The Dof members could play functions in the regulation of secondary metabolic procedures, under biotic and abiotic stress tolerance^22^. Abscisic Acid-Insensitive 5-like protein 4 isoforms play a major role in abiotic stress tolerance in plants^23^. The aims of the study are to determine the nature of heat stress tolerance in cucumber plants morphologically, biochemically, and molecularly besides identifying expression patterns for candidate genes at different growth stages.

## Materials and methods

### Experiment design, growth, and climate conditions

Experiments were conducted under greenhouse conditions at Horticultural Research Departments of Horticultural Research Institute, Agricultural Research Center. The genotypes were obtained from the inbreeding cucumber program at Cross-pollinated Vegetable Research Department, Horticulture Research Institute, Agriculture Research Center, Egypt. Seedlings of P_1_, P_1_×P_2_, and Barracuda (Aggrotech seed company) were transplanted to a greenhouse, on May 9th into two rows within the bed (row 7 m long and 1.0 m in width). The space was 0.5 m between the plants. The experimental unit consisted of 14 plants in each row. The natural thermal stress temperatures above 38 °C during May, June, and July recorded in Table 1 by BST-DL13 (B091BRMT7C).

**Table 1:** Actual monthly maximum air temperatures (°C) during different growth stages in two growing seasons.

Month	Growth stages	First season		Second season	
		Max. temperature	No. stress hours˃38 °C	Max. temperature	No. stress hours˃38 °C
May	Vegetative growth	45 °C	150 h	46 °C	162 h
June	Vegetative growth and flowering	45.6 °C	180 h	46.3 °C	177 h
July	Flowering and fruiting	47.3 °C	210 h	47 °C	216 h
Average	Around all stages	45.96 °C	180 h	46.43 °C	185 h

No. stress hours= duration of heat stress per day × number of days per month.

All methods were performed in accordance with the relevant guidelines/regulations/legislation.

### Morphological traits

Morphological data were recorded on 20 plants to assess 14 horticultural traits under heat stress conditions: main stem length (MSL; cm) at the end of the season; internode length (IL; cm); the number of lateral branches (No. LB) for the primary 50 cm; leaf area(LA;cm^2^); days to first female flower opening (DFFO); the number of female flowers per node (NFF/node); average fruit weight (FW; g); average fruit length (FL; cm); average fruit diameter (FD; cm); the ratio between fruit length and fruit diameter (FL/D); the quantity of early fruits (No. EF) was measured for 20 days from harvest started; early fruit weight (EFW; kg); the number of total fruits (No. TF) were measured 3 times weekly for 4 weeks from the primary harvest; a total of fruit weight (TFW; kg) was measured 3 times weekly for ten weeks from the primary harvest. Nine importance descriptive traits, nature of growth, leaf color, flowering nature, fruit color, pedicle fruit, fruit ribbed, bitterness, fruit neck, and spines were determinate. Fruit descriptors were evaluated 20 plants per genotype under natural heat stress conditions.

### Heat injury index

This assessment was administered to assess the heat tolerance mechanisms of the foremost tolerant genotypes, the parental line P_1_, and hybrid (P_1_ × P_2_) compared with the foremost sensitive genotype, Barracuda F1. Heat stress symptoms clearly obvious during the second month after transplanting. The heat injury index (HII): heat tolerance performance for every plant was recorded. The heat injury index for leaves (HIIL%) was classified into sex degree according to the familiar dryness area of the three entire leaves (8th–10th), and the heat injury index for female flowers (HIIF) entire on female flowers/nodes (5th–10th). HIIL% was as follow: 0 = no injury on the 8th to10th leaves; 1 = only ends of the 8th to 10th leaves were dried; 2 = 1/3 of the 8th to 10th leaves were dried; 3 = 1/2 of the 8th to 10th leaves were dried; 4 = over 2/3 of the 8th to 10th leaves were dried; 5 = the whole 8th to 10th leaves were dried. While HIIF% was as follows: 0 = no injury on the 5th to 10th female flowers; 1 = only 1 of the 5th to 10th female flowers were dried; 2 = 1/3 of the 5th to 10th female flowers were dried; 3 = 1/2 of the 5th to 10th female flowers were dried (brownish); 4 = quite 2/3 of the 5th to 10th female flowers were dried; 5 = the whole 5th to 10th female flowers were dried. After heat stress treatment, phenotypic data of the heat injury for leaves and female flowers index were recorded in population. The heat injury index (HII) was calculated according Wei et al.^24^ using the formula as follows:

HII = (0 ×S0 + 1 × S1 + 2 × S2 + 3 × S3 + 4 × S4 + 5 × S5)/ (5 × N) ×100,

S0–S5 indicates the number of plants corresponding to each grade. N indicates the total number of plants. For each experiment, the HII of each line was calculated by taking the average of the HII in five replicates^25^.

### Proline estimation

The free proline content was quantified utilizing the method described by Bates et al.^26^. 500 mg samples that freeze and dried were homogenized in 5 mL of three (w/v) sulphosalicylic acids. The homogenate was filtered through paper (Whatman, No.1). The filtrate was mixed with a ninhydrin acid reagent (2% v/v) and acetic acid. In a very boiling water bath for 45 min at 100 °C for 1 h, the mixture was placed. Then, 4 mL of toluene was added and maintained in the tubes for 20 s. To stop the reaction, the tubes were placed in crushed ice. The free proline was detected spectrophotometrically at a 520 nm wave against the reagent blank.

### Chlorophyll estimation

The total chlorophyll content was defined within the fresh leaves as described by Lichten-thaler and Buschmann^27^ procedure. 0.5 g of leaf samples were milled in 80% acetone (Sigma-Aldrich Co. LLC, Saint Louis, MO, US). The samples were centrifuged at 1100 × *g* for 8 min at 4 °C. After, that, the supernatant part was analyzed employing a spectrophotometer (Helios UVG1702E, Cambridge, UK). The values of the total chlorophyll were described in mg. g−1 FW.

### Plant hormones estimation

The content of gibberellin (GA3) and abscisic acid (ABA) within the cucumber leaves was assessed at 14, 30, and 45 days after transplanting using the tactic noted by Fales et al.^28^. Briefly, freeze-dried cucumber leaves were mild to a fine powder. 10 mg of fine powder was washed 3 times with 80% methanol (v/v) and 2,6-bis (1,1-dimethylethyl)-4- phenol at 4 °C within the dark. The extract was centrifuged at 4000 rpm, and the supernatant was adjusted to pH 8.6, so the residues were extracted twice with an equal volume of pure ethyl acetate. The mixed supernatant with ethyl acetate extracts was dehydrated over anhydrous sodium sulfate and filtered. Under a vacuum at 35 °C and redissolved in 1 mL absolute methanol, the filtrated supernatant was evaporated. The ultimate extract was filtrated and dehydrated^28^. The quantification of abscisic acid (ABA) and gibberellin (GA3) was determined using pure standards of the hormones and a Microsoft program to calculate the concentrations of the identified peaks. Regarding, brassinosteroid assay, leaves samples were ground with 10 mL of 80% methanol extraction solution that contain 1 mM butylated hydroxytoluene. The mixture was incubated for 4 h at 4 °C. For 10 min at 3500 *g*, the samples were centrifuged. The supernatants were filtered through a C18-Sep-Pak cartridge (Waters, Milford, MA, United States), and the efflux was collected and dried. The mixture dissolved in 2 mL of PBS containing 0.1 % (v/v) Tween 20 and 0.1% (w/v) gelatin (pH 7.5). The samples were analyzed via indirect enzyme-linked immunosorbent assay. The calibrating samples (epibrassinolide, CAS: 72962-43-7) or test samples (150 μL/well) were put in wells of the plate with the immobilized antibodies. Plates were placed at 37 °C for 30 min. The horseradish peroxidase (HRP)-conjugate (150 μL) was placed within the wells and placed at 37 °C for 30 min. Then, removed the liquid from the wells, and washed plates quadrupled with washing buffer. Added TMB solution (containing H2O2) to the wells and placed the plates at 37 °C for 20 min. Quenched the reaction by adding 2 mol L-1 H2SO4 (50 μL) into each well. Calculated the concentration in keeping with the calibration curve. The determination of BR was done at 450 nm optical absorbance according to Swaczynov et al.^29^.

### Antioxidant enzymes estimation

The antioxidant enzymes' activity was estimated in 0.5 g fresh leaf samples that were collected at 14, 30, and 45 days and ground in 5 ml sodium phosphate buffer (pH 7.6) for 10 min at 4 °C, including 1 mM EDTA and 4% (w/v) PVP and then incubated. The homogenate was then centrifuged (12,000g) for 15 min at 4 °C, and the upper phase supernatant was used for subsequent assessment of enzymes^30^. General activity of superoxide dismutase (SOD, EC 1.15.1.1) was estimated spectrophotometrically at 560 nm according Giannopolitis and Ries^31^ Regarding the POD (EC 1.11.1.7) determination was done according the protocol of Hernandez et al.^32^. POD absorbance was recorded spectrophotometrically at 470 nm. Catalase activity (CAT, EC 1.11.1.6) was determined by the enzymatic decomposition of H_2_O_2_ at 240 nm^33^.

### Identification of genes

The protein sequences of genes involved in ABA, GA3, BR, and AOX metabolism and transport in Cucumis sativus, Cucumis melo, Cucurbita moschata, Cucurbita pepo, Cucurbita pepo subsp. pepo, Arabidopsis thaliana, Cucurbita maxima, Luffa aegyptiaca, Corchorus capsularis, and Momordica charantia were downloaded from http://www.ncbi.nlm.nih.gov/Genbank/l, http://cucurbitgenomics.org/organism/16, and http://www.uniprot.org/. These sequences were used as queries for protein blast analysis against the cucumber reference genome database (Cucumber (Gy14) v2 Genome, Cucurbit Genomics Database (CuGenDB). MEGA X software was used to draw phylogenetic trees. Clustal W tool was used to align protein sequences and neighbor-joining method with 1000 bootstrap replicates to construct trees^34,35^. The prediction of the candidate genes was based on the gene annotation in the reference genome of cucumber “Gy 14 V2.0” http://cucurbitgenomics.org/organism/16. Genes associated with heat stress tolerance such as ABA, GA3, BR, and AOX were selected. Based on the resequencing data of P_1_, P_1_×P_2_ and barracuda F_1_ polymorphisms of the chosen genes between “P_1_”, “P_1_×P_2_” and barracuda F_1_ were tested. The 12 genes were along with their accession numbers, their genomic lengths, coding sequence lengths, protein sizes, and isoelectric points (pIs) and Mw (Da), were retrieved from two online tools, (i) http://cucurbitgenomics.org/organism/16 and (ii) ExPASy. http://web.expasy.org/computepi/databases.

### RT-qPCR expression analysis

Genes linked to the ABA, GA3, BR, and AOX metabolism and transport were selected from the cucumber genome database (Cucumber, Gy14) v2 Genome (Cu Gen DB). To test the expression patterns of selected genes in cucumber, leaves of “P_1_”, “P_1_×P_2_” and “barracuda F_1_” were collected at 14, 30, and 45 days after transplanting, and RT-qPCR was performed. consistent with the manufacturer’s instructions, total RNA was extracted using RNA Kit (Tiangene, China). 1% agarose gel was accustomed check RNA degradation and contamination. RNA quality and integrity were checked via Nano Drop ND-1000 spectrophotometer (Thermo Scientific, Wilming-ton, DE, USA) and an Agilent 2100 Bioanalyzer (Agilent Technologies, CA, USA). Extracted RNA was used for the cDNA synthesis for RT-qPCR, with M-MLV polymerase (Promega, USA). Specific primers for every gene are listed in Table S1. Actin1 was applied as a reference gene for normalizing gene expression values^36^. Three independent biological replicates were used for gene expression analysis, the complete data were analyzed using the 2-ΔΔCt method^37^.

### Experimental design and statistical analysis

The experiments were designed in randomized complete block design with five replicates. Data were statistically analyzed, using analyses of variance (ANOVA) with the Stat soft statistical package (MSTATC) software program (Michigan State University, East Lan-sing, MI, U.S.A.). Probabilities of significance among genotypes compared with the least significant difference L.S.D. (P ≤ 0.05) according to Gomez and Gomez^38^.

## Results

### Morphological traits

**Figure 1:**
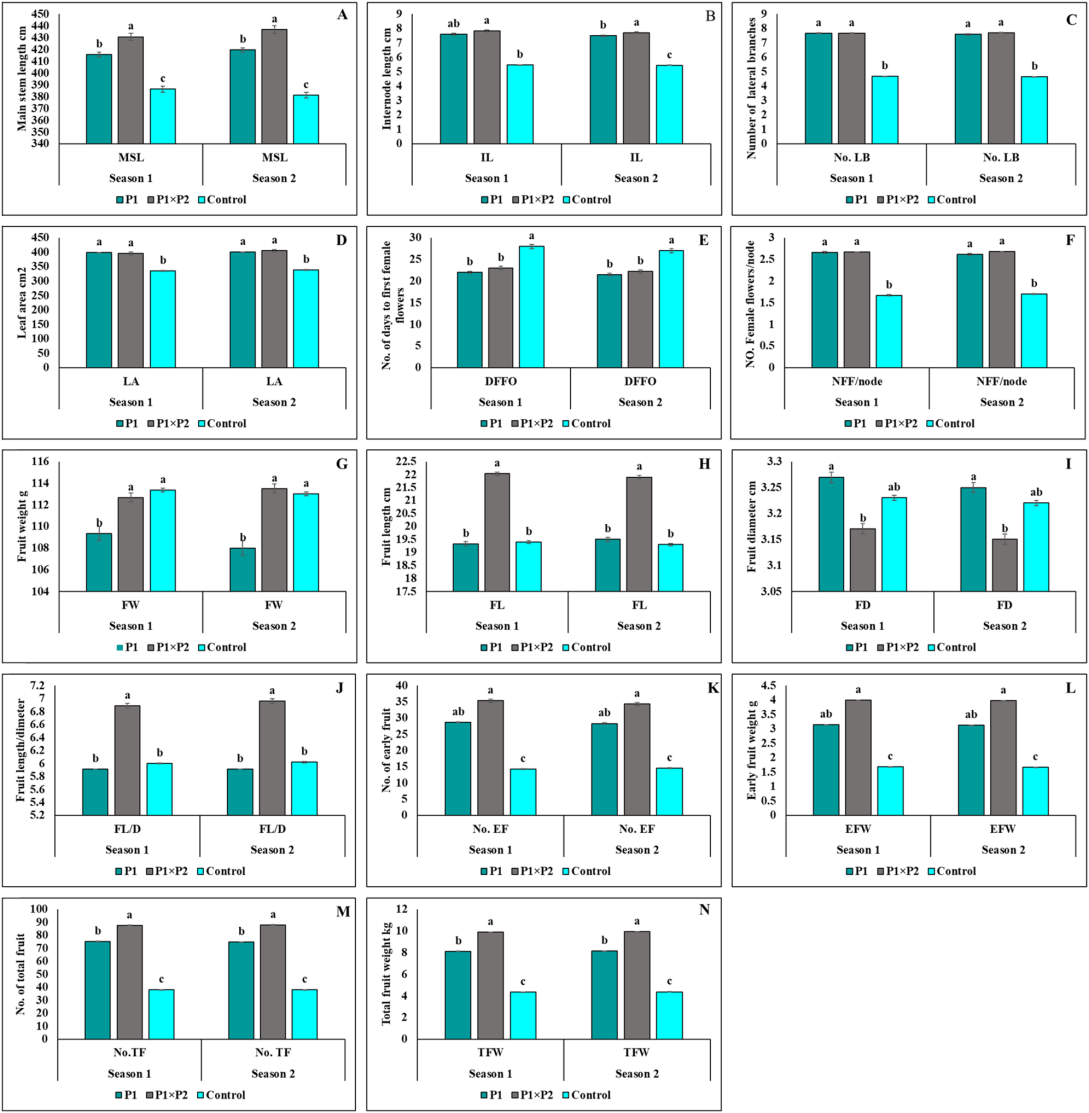
(**A**–**N**) Mean performance of all cucumber genotypes for vegetative traits (**A**) main stem length(cm); (**B**) internode length (cm); (**C**) number of lateral branches; (**D**) leaf area (cm^2^), flowering traits (**E**) number of days to first female flower opining, and (**F**) number of female flowers per node, fruits traits, (**G**) fruit weight (g); (**H**) fruit length (cm), and (**I**), Fruit diameters(cm); (**J**) the ratio between fruit length and diameter and yield component traits, (**K**) number of early fruit; (**L**) early fruit weight (kg); (**M**) number of total fruit, and (**N**) total fruit weight (kg). Means (SE) followed by the same letter are not significantly different at *p* ≤0.05 LSD.

There were significant variations in mean across P_1_, P_1_×P_2_, and Barracuda F_1_ for horticultural estimated traits under natural heat conditions (Fig. 1A–N). Parents 1 was superior in the vegetative traits, earliness, and yield components, however, there have been quite substantial variations between P_1_, P_1_×P_2_, and the control. The P_1_×P_2_ had the highest mean value for vegetative traits, followed by P1. They have the highest stem length (437 and 415.667 cm), the longest node (7.83 and 7.6 cm), many lateral branches (7.7 and 7.667), and also the biggest leaf area (405 and 399.333 cm^2^). The P_1_ and P_1_×P_2_ excel not only in terms of vegetative traits, but also in terms of flowering and yield component features side to side heat tolerance. The inbred line 1 was the earliest blooming inbred line, as evidenced by its early and total yield productivity of 3.143 and 8.147 kg/plant, respectively, when put next to control. The cross (P_1_×P_2_) surpassed the commercial hybrid's altogether measured attributes, generating 9.922 kg/plant and 88 fruits/plant, compared with 4.37 kg/plant and 38.09 fruits/plant for the control with note clear decrease in productivity under heat stress conditions.

### Heat-injury index

**Figure 2:**
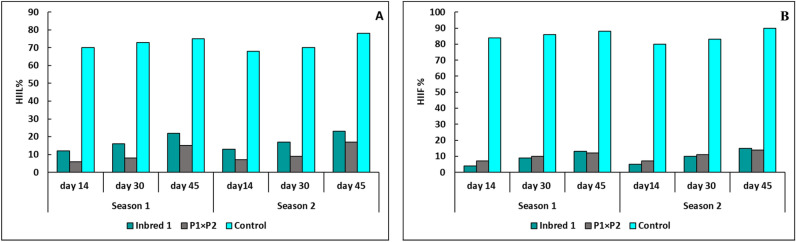
The average of heat injury index for leaves HIIL (**A**) and flowers HIIF% (**B**) of parent P_1_ (HT), hybrid P_1_×P_2_ (HT), and control (HS) at 14, 30, and 45 days for two season.

**Figure 3:**
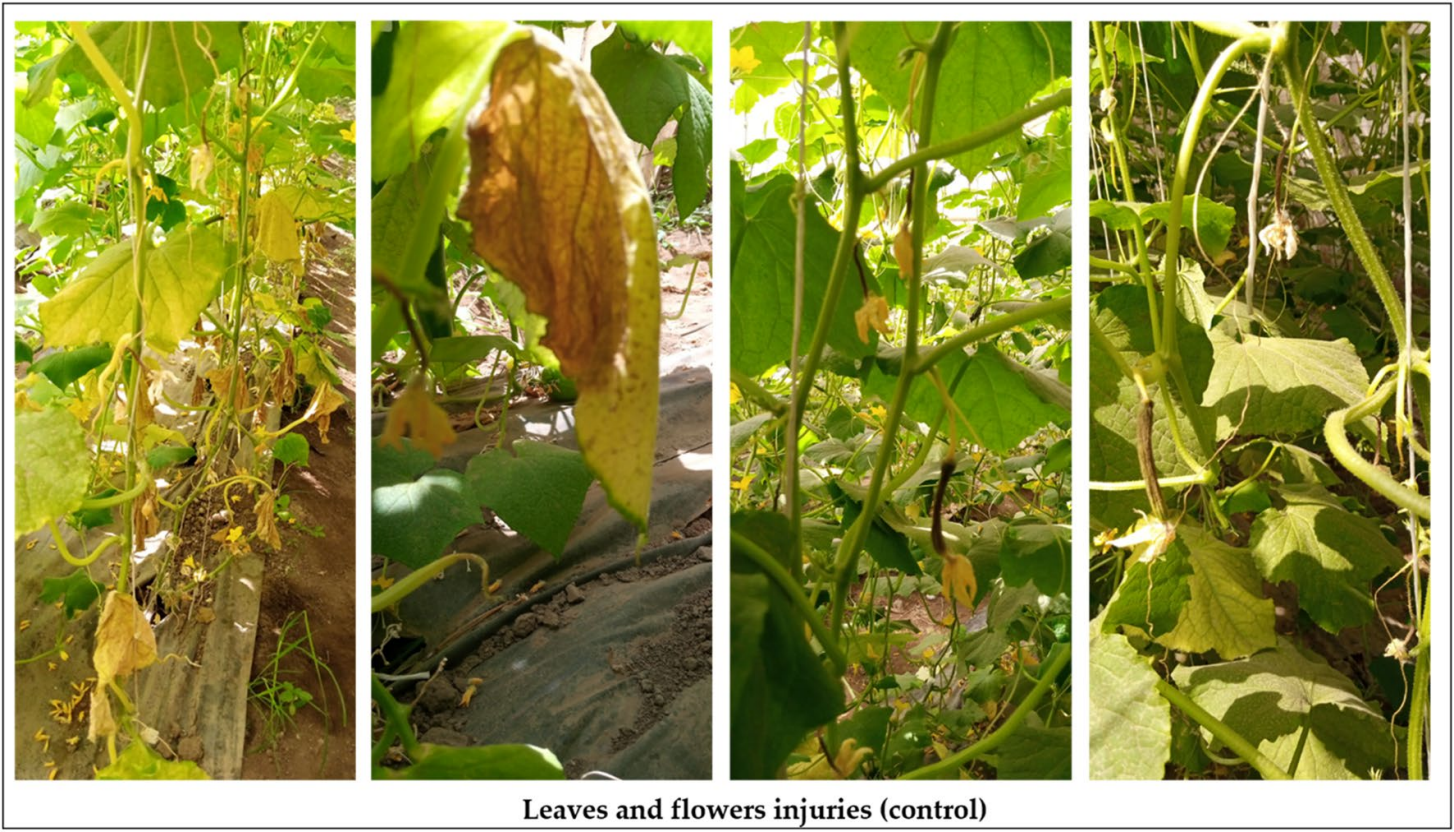
The heat injuries symptoms on leaves and flowers in sens**i**tive plants (HS).

**Figure 4:**
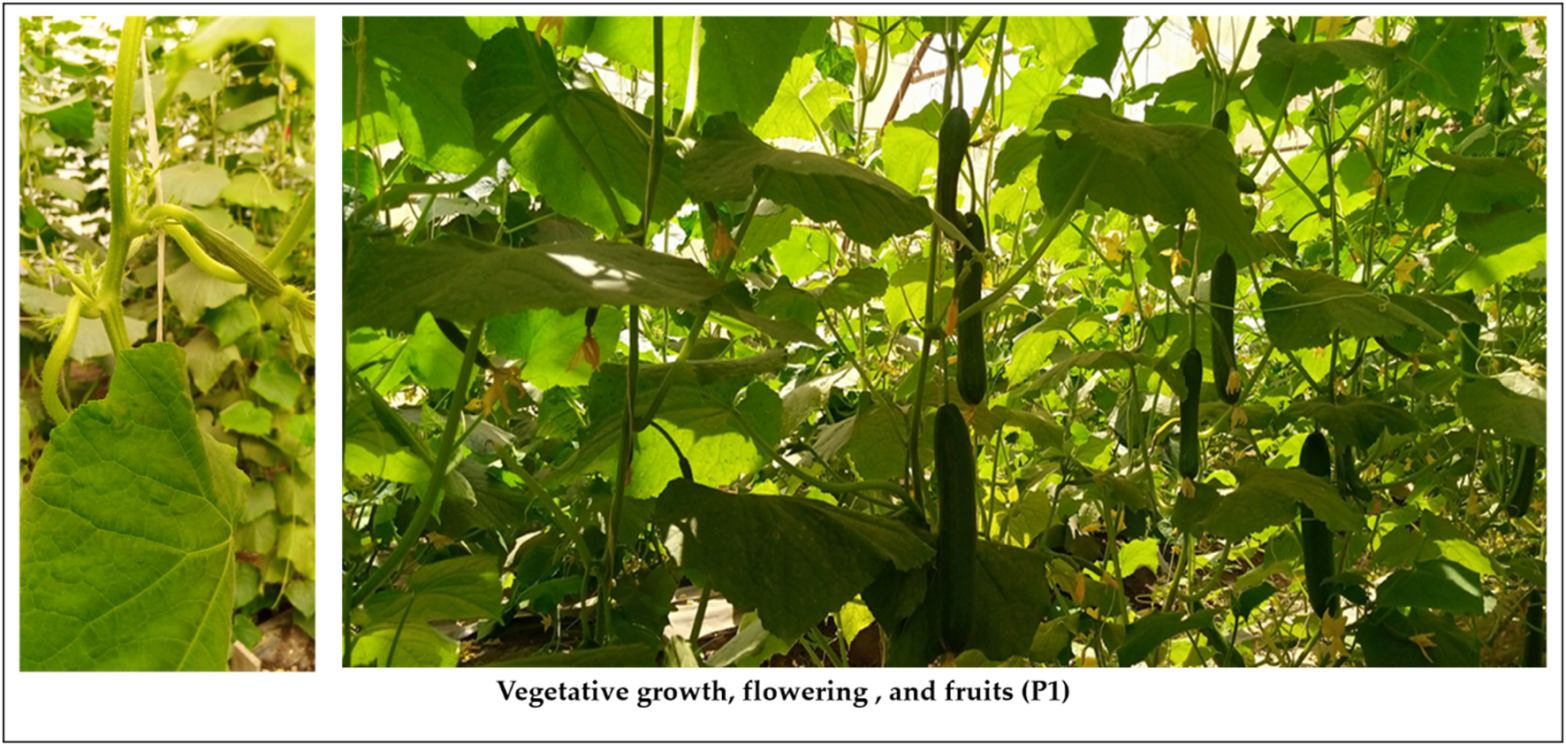
The heat stress tolerance on leaves and flowers in parent P_1_ plants (HT).

**Figure 5:**
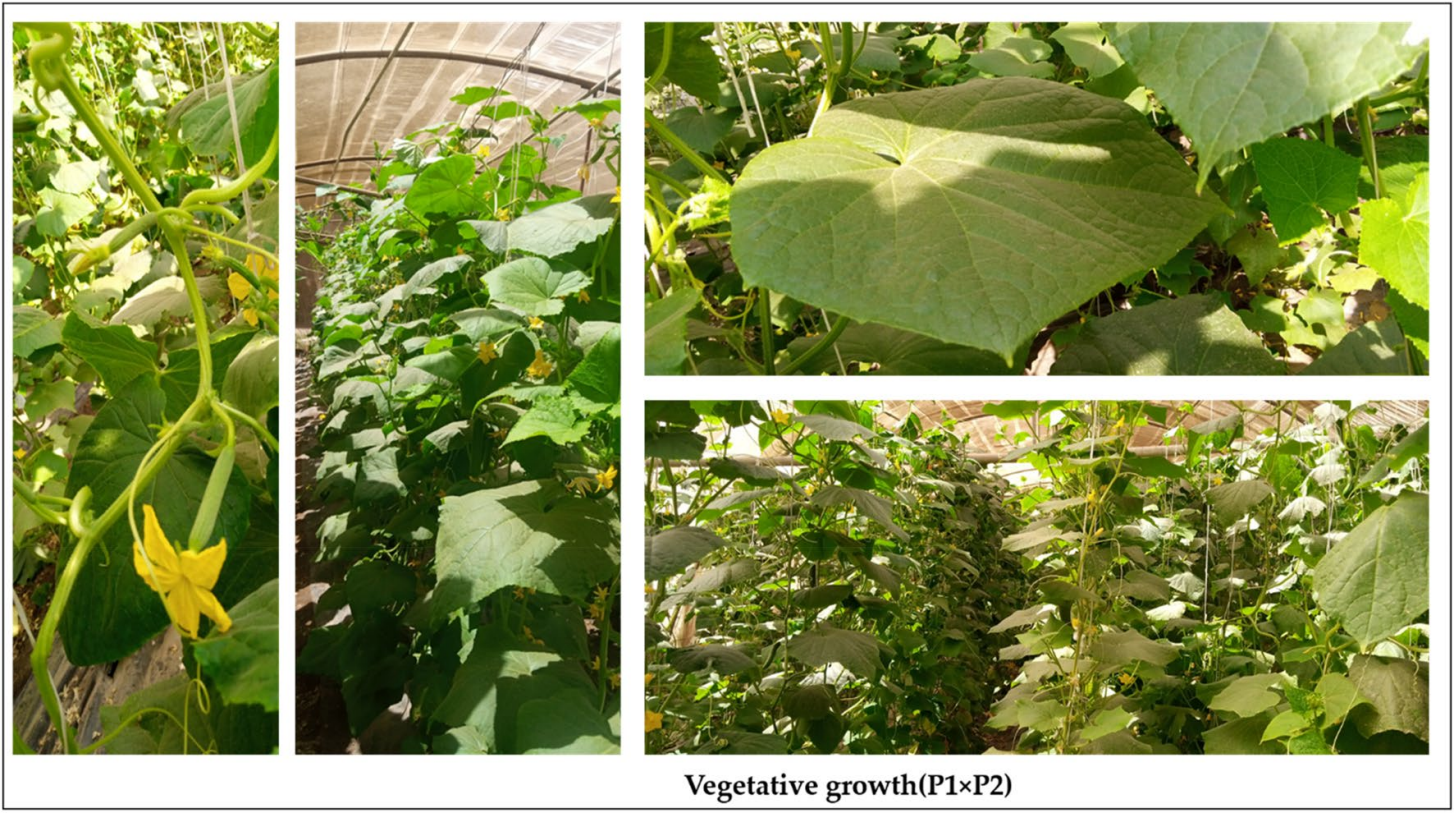
The heat stress tolerance on leaves and flowers in hybrid plants, P_1_×P_2_ (HT).

The parental line P_1_, hybrid P_1_× P_2_, and Barracuda F_1_ were grown in the traditional un control greenhouse for two summer seasons. In 2020 and 2021, the plants were exposed to natural heat stress temperature higher for three months. Symptoms of heat injury become obvious after 14 days from transplanting. The heat injury index was ranged into six stages based on leaves dryness and female flowers failed. The heat injury index was used to refer to the heat stress in each plant among genotype (Fig. 2). The Results proved that “P_1_” and “P_1_× P_2_” showed highly tolerance to heat stress, while, barracuda F_1_, recorded sensitivity to heat stress in two seasons (Figs. 3, 4 and 5). The genotypes “P_1_” and “P_1_× P_2_” grew normally, and heat injury index for leaves showed no significant damage in two seasons. The HIIL% did not excessed 25% for P_1_, and P_1_× P_2_ compared with higher than 70 % for barracuda F_1_ at 14, 30, and 45 days in two seasons. However, HIIF % for P_1_ and P_1_× P_2_ were less than 20% which showed no clearly damaged observed on female flowers in contrast HIIF% for barracuda F_1_ reached to 90% which showed completely female flowers dead and failed to complete in fruit shape in some plants (Figs. 3).

### Total chlorophyll and proline content

**Figure 6:**
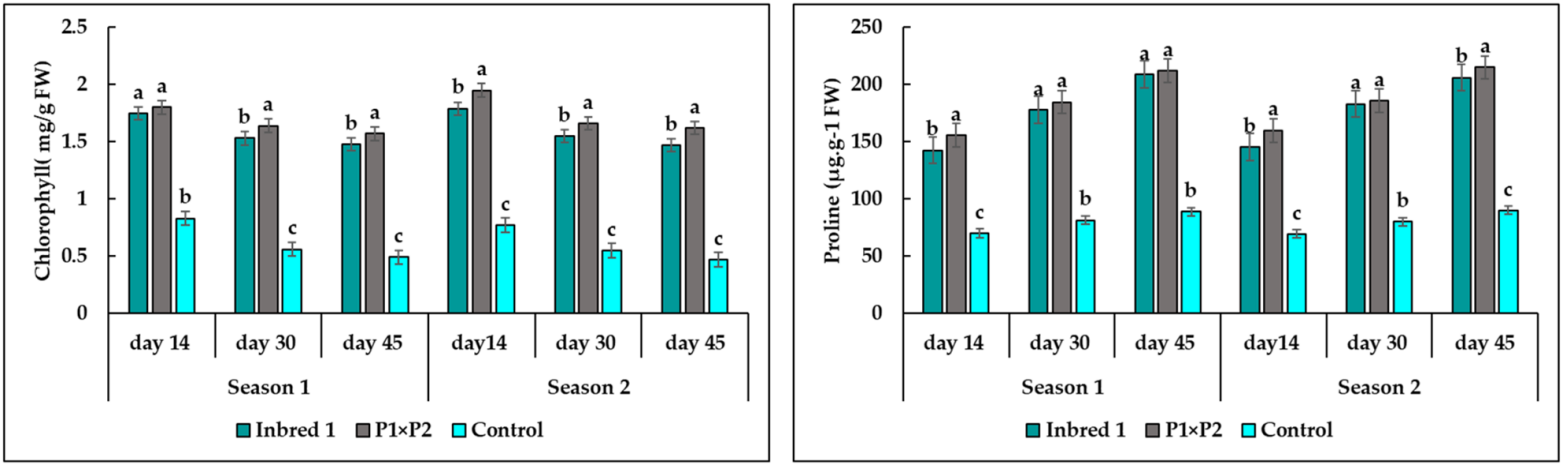
The chlorophyll and proline content in leaves of parent P1 (HT), hybrid P1×P2 (HT), and control (HS) for means at 14, 30, and 45 days of two season. Means (± SE) followed by the same letter are not significantly different at *p* ≤0.05 (LSD test).

Total chlorophyll was estimated in heat-tolerant genotypes (P_1_ and P_1_×P_2_) and heat-sensitive control in two seasons (Fig. 6). It was noticed that after 14 days from trans-planting under heat stress conditions, heat tolerant (HT) plants have identical total chlorophyll, which non-significant decrease gradually from 30 to 45 days after transplanting, but this decrease did not cause damage to leaves, flowers, and fruit phenotype. However, heat stress reduced chlorophyll content in control plant (HS), and the injury was irrecoverable in HS plants after 45 days. The proline content in leaves was increased under heat stress conditions in heat tolerant genotypes (P_1_ and P_1_×P_2_) and heat sensitive plants (control). The proline content of P_1_, P_1_×P_2_, and F_1_ Barracuda leaves quickly increased with heat stress conditions at 14, 30, and 45 as shown in Fig. 6. The proline content of HS leaves also increased during that time. There are significant differences between the proline content in P_1_ and P_1_×P_2_ compared with F_1_ Barracuda. P_1_ and P_1_×P_2_ leaves had an increase in proline content, without clear differences. The results showed that the proline content increased gradually in both HT and HS cucumber plants. However, the proline concentration in HT plants was significantly higher than the HS cucumber, which reached the highest level in HT plant at 45 days without significant differences between P_1_ and P_1_×P_2_ (HT).

### Antioxidant enzymes analysis

**Figure 7:**
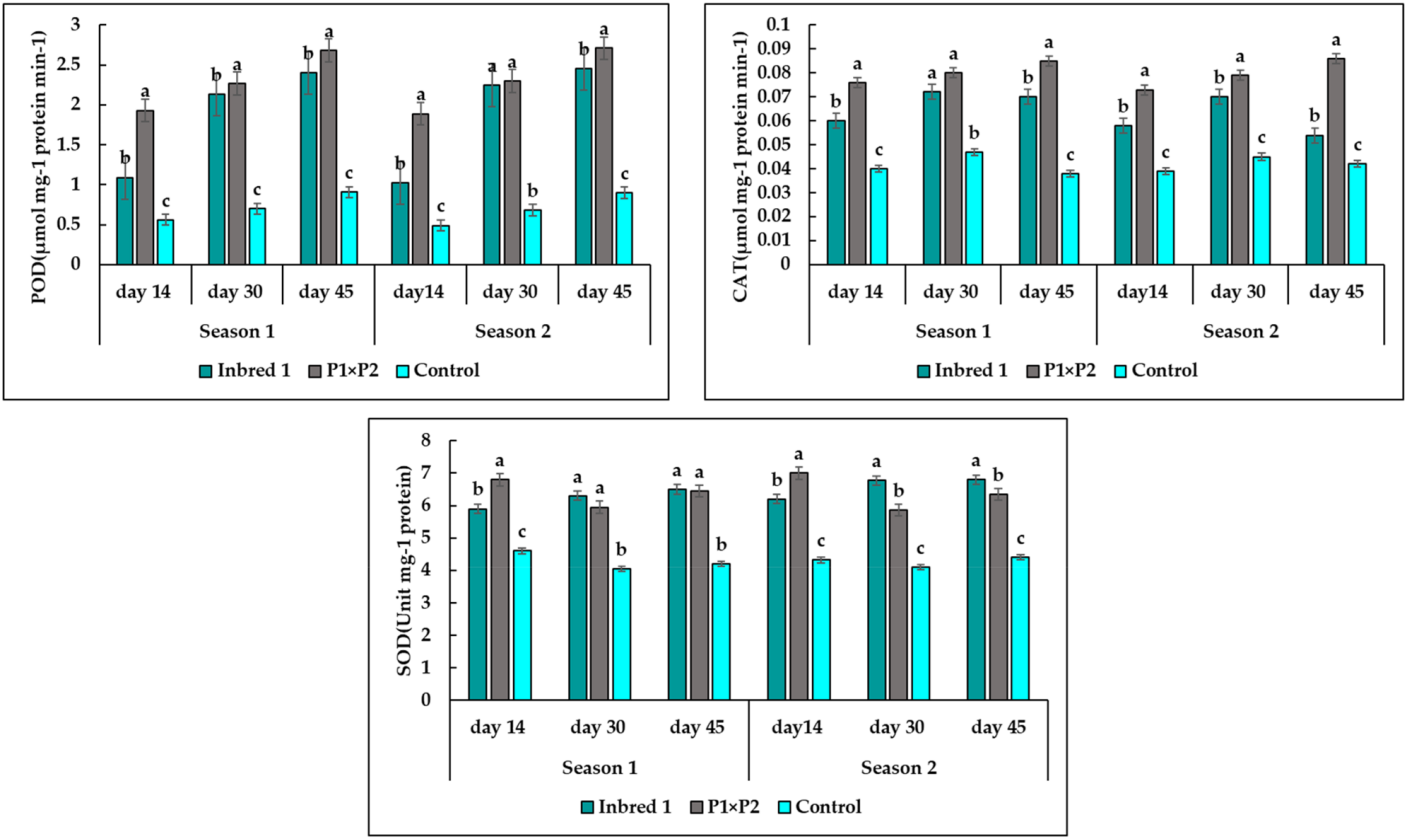
Changes in POD (peroxidase), CAT (catalase), and SOD (superoxide dismutase) enzyme activities in heat-tolerant HT (of parent P1, hybrid P1×P2) and heat sensitive HS (control) cucumber leaves under heat stress condition at 14, 30, and 45 days. Means (± SE) followed by the same letter refer to insignificant differences at *p* ≤0.05 (LSD test) of two season.

Heat stress conditions produced obvious enhancement in enzyme activities of POD, CAT, and SOD in P1 and P1×P2 as heat tolerant (HT), and F1 Barracuda as heat sensitive (HS) (Fig. 7). For both the healthy and injured leaves, enzyme activities of POD, CAT, and SOD had raised gradually from 15 days to 45 days after transplanting under natural heat stress conditions in both seasons. The activities of POD, CAT, and SOD were higher in HT leaves than in HS leaves. The results showed that CAT, POD, and SOD in HT under heat stress conditions were a higher concentration than HS at 14, 30, and 45 days from transplanting. The results also illustrated significant differences between P_1_ and P_1_× P_2_ (HT) in AOX concentration at most samples stages towards the hybrid P_1_× P_2_.

### Endogenous phytohormones

**Figure 8:**
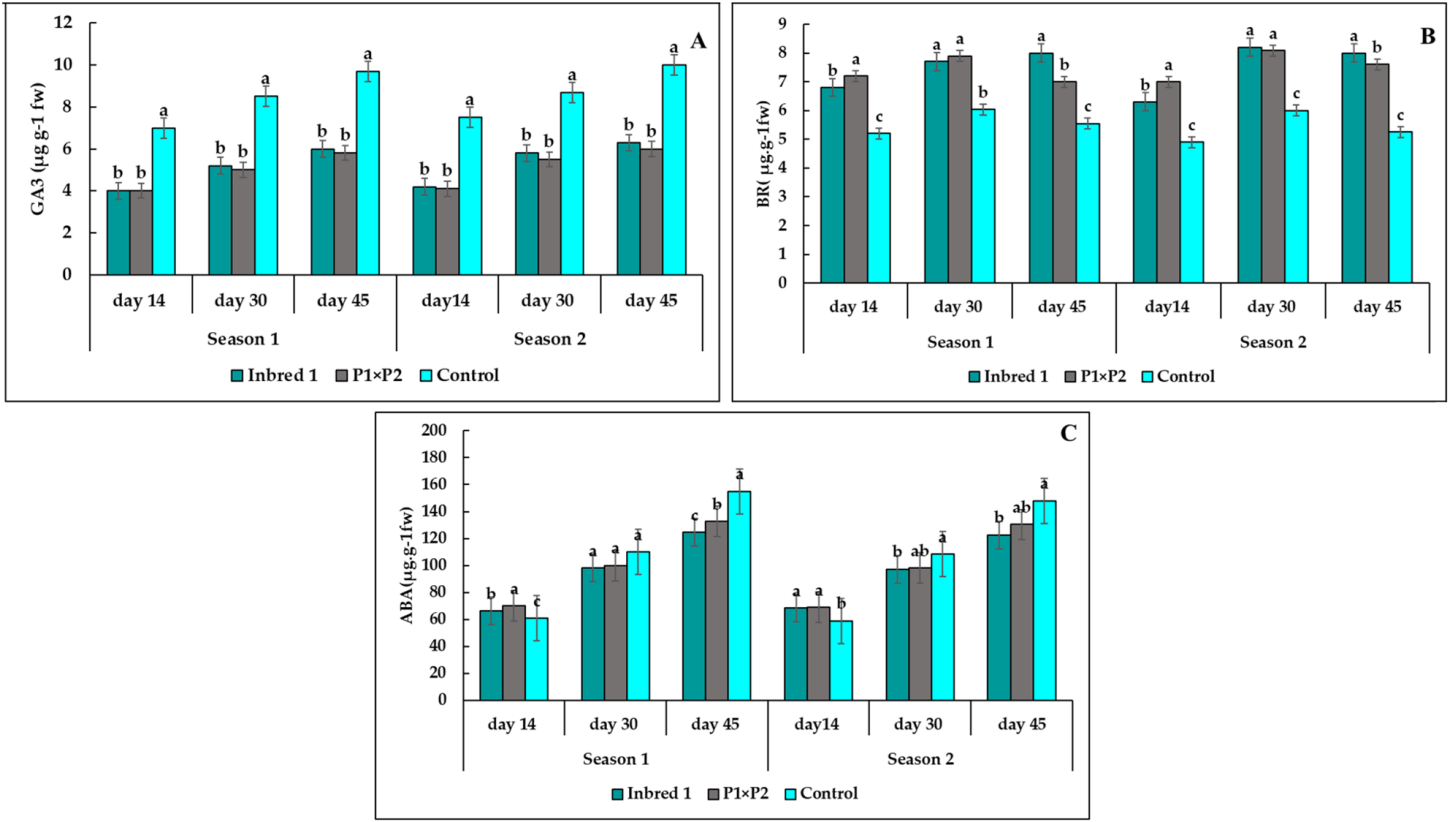
Changes in GA3 (Gibberellic acid), BR (Barrsinostroid), and ABA (Abscisic) enzyme activities in heat-tolerant HT (parent P_1_, hybrid P_1_ × P_2_) and heat sensitive HS (control) cucumber leaves under heat stress condition at 14, 30, and 45 days. Means (± SE) followed by the same letter refer to insignificant differences at *p* ≤0.05 (LSD test) of two season.

The High temperature stress affected on hormone synthesis in all genotypes as presented in Fig. 8. The HT and HS plants had similar levels of abscisic acid (ABA) at 14, 30, days while the control plants (HS) had the highest significant concentration at 45 days in both seasons. Furthermore, the HS plants had the highest GA3 concentration at 14, 30, and 45 days. In contrast, the P_1_ and P_1_×P_2_ (HT) had the higher concentration of gibberellin (GA), and brassinosteroid (BR) than HS at all sample stages. In most cases P_1_ had the higher significant phytohormones contents than the hybrid P_1_ × P_2_.

### Phylogenetic analysis of selected genes

**Figure 9:**
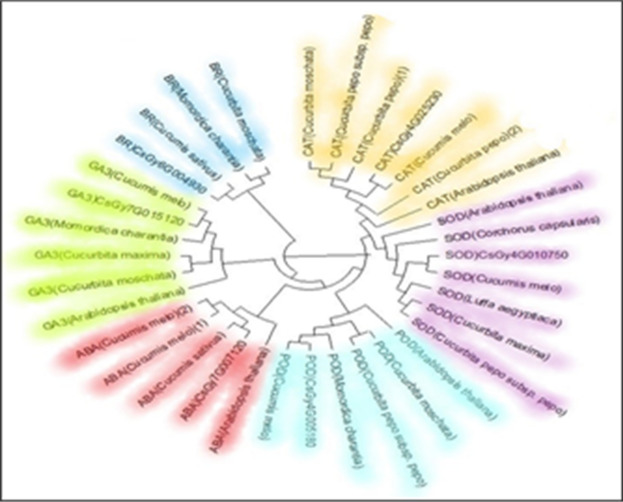
Maximum likelihood trees genes Note: involved in CAT, POD, SOD, ABA, GA3 and BR metabolism and transport with those from *Cucurbita moschata*, *Cucurbita pepo*, *Cucumis sativus*, *Cucumis melo*, *Cucurbita pepo* subsp. *pepo*, *Arabidopsis thaliana*, *Cucumis melo*, *Cucurbita maxima*, *Luffa aegyptiaca*, *Corchorus capsularis*, and *Momordica charantia*. The protein sequences were used to make a phylogenetic tree by the neighbor-joining method.

Cucumber Genome Database was explored for Blast P searches in Fig. 9, using *Cucumis sativus, Cucurbita moschata, Cucurbita pepo, Cucumis melo, Cucurbita pepo subsp. pepo, Arabidopsis thaliana, Cucurbita maxima, Luffa aegyptiaca, Corchorus capsularis*, and *Momordica charantia*. Protein sequences of genes associated with ABA, GA3, BR and AOX were used as inquiry and allowed candidates to discover candidate genes in the case of cucumber. Cucumber genes having high homology are inserted in (Table S2).

### Gene expressions RT-qPCR analysis

**Figure 10:**
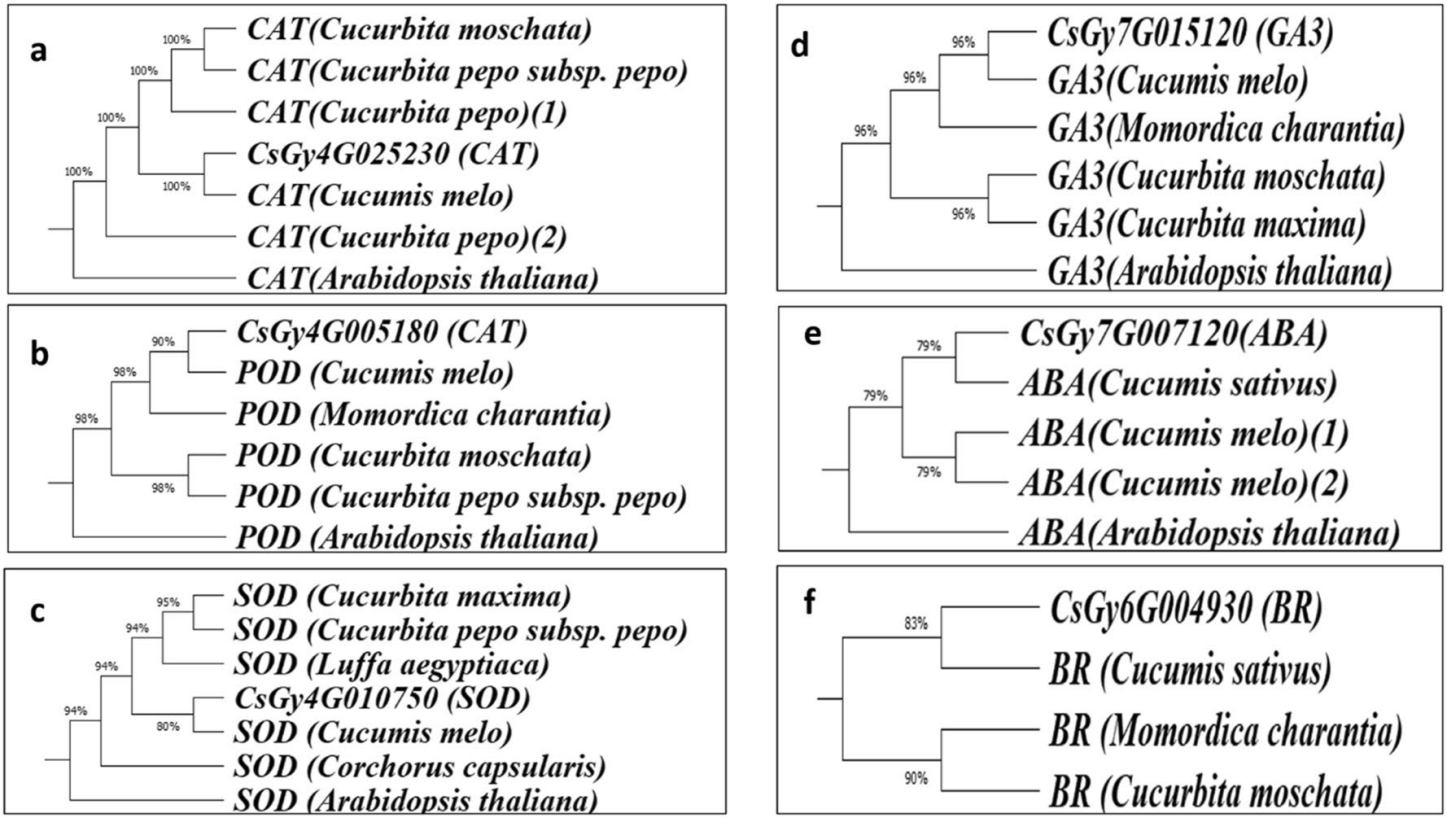
Maximum likelihood trees genes Note: involved in CAT, POD, SOD, ABA, GA3 and BR metabolism and transport with those from *Cucurbita moschata*, *Cucurbita pepo*, *Cucumis sativus*, *Cucumis melo*, *Cucurbita pepo* subsp. pepo, *Arabidopsis thaliana*, *Cucumis melo*, *Cucurbita maxima*, *Luffa aegyptiaca*, *Corchorus capsularis*, and *Momordica charantia*. The protein sequences were used to make a phylogenetic tree by the neighbor-joining method.

The temporal expression pattern of the twelve candidate genes after 14, 30, and 45 days from transplanting, was discussed in “HT” and “HS” (Table 2). The results showed that the expression of phytohormone genes CU-Gibberellin-1 (*CsGy7G019290* and *CsGy7G015120*) in “HS” was significantly higher than “HT” at 45 days and the gene expression level was increased from 30 to 45 day in “HT”. Transcript levels of genes were associated with heat stresses they regulate important biochemical hormones. CU-bar-1 (*CsGy6G004930*) and (*CsGy6G029150*), had higher expression controlling BR signaling in (HT) cucumber at 14, 30, and 45 days after transplanting during the heat stress in both seasons. The expression level of CU-ABA-1 (*CsGy7G007120, CsGy2G018910*) in “Barracuda F_1_ “HS” was significantly higher than “HT” at 30 and 45 days. While, the expression levels of antioxidant enzymes, CU- CAT-1 (*CsGy4G025240* and *CsGy4G025230*) in “HT” were higher than “HS” at 14, 30, and 45 days and the highest expression level was on 30 and 45 days. The expression levels of CU-POD-1 *CsGy6G015230* and *CsGy4G005180* had significant differences between “HT” and “HS” toward HT at 14, 30, and 45 days. The CU-POD-1 (*CsGy4G005180*) controlling POD synthesis had a highest expression in HT as compared to HS cucumber followed by CU-SOD-1 (*CsGy4G010750*) at 14, 30, and 45 days in two seasons (Fig. 10).

**Table 2:** Analysis of candidate genes related to cucumber heat tolerance.

Gene ID	Location	Gene function annotation
*CsGy7G007120*	Chr7: 5133539. 5136186 (+)	ABSCISIC ACID-INSENSITIVE 5-like protein 4 isoform X1
*CsGy2G018910*	Chr2: 28418722. 28422428 (−)	ABSCISIC ACID-INSENSITIVE 5-like protein 5
*CsGy7G015120*	Chr7: 19103979. 19105606 (+)	Gibberellin receptor GID1B
*CsGy7G019290*	Chr7: 21920567. 21922180 (−)	Gibberellin 3-beta-dioxygenase 1-like E1
*CsGy6G004930*	Chr6: 4561902. 4563149 (+)	Probable carbohydrate esterase At4g34215
*CsGy6G029150*	Chr6: 27853373. 27855339 (1.97 Kb)	Dof zinc finger protein DOF5.7
*CsGy4G025240*	Chr4: 30694676. 30698026 (+)	Catalase
*CsGy4G025230*	Chr4: 30688897. 30692995 (+)	Catalase
*CsGy6G015230*	Chr6: 13799140. 13800508 (−)	Peroxidase
*CsGy4G005180*	Chr4: 3732444. 3735094 (−)	Peroxidase
*CsGy1G026400*	Chr1: 24981634. 24984716 (+)	Superoxide dismutase [Cu-Zn]
*CsGy4G010750*	Chr4: 9907339. 9916663 (+)	Superoxide dismutase [Cu-Zn]

## Discussion

The heat tolerance in cucumber has become necessary for a stable life cycle, including, plant growth and production. So, one of the foremost critical purposes of the breeding programs yielding high thermos-tolerant inbred lines and hybrids^3,39^. Previous investigations utilized plant height, stem diameter, leaf area, female flower number^40^, and yield loss as thermos tolerance in cucumber^9^. In the current study, the heat injury index for leaves and the female flowers were estimated to indicate the heat tolerance ability at three growing stages 14, 30, and 45 days from transplanting. Moreover, the study was conducted in a plastic greenhouse for two seasons, where cucumber has grown under a natural temperature of around 45 °C, which actually badly influenced production. The obtained results documented the tolerance of P_1_ and P_1_×P_2_ for natural heat stress conditions due to their outstanding traits such as growth, fruit quality, earliness, and early and total yield^19,39^. Plants use complicated various of mechanisms, which implicate numerous interaction pathways, to treat stress. A number of these mechanisms contain the regulation of plant hormones, transcription factors, and miRNA; as the transmission and power of signal factors^18,41^. Within the current study, two heat tolerant and one heat-sensitive cucumber genotypes were selected to spot their physical responses to high-temperature exposure. Organic phenomenon and physiological changes analyses were performed to look at the responses of the three cucumber genotypes to heat stress following 14, 30, and 45 days after transplanting under natural heat exposure. The result revealed that the two cucumber genotypes displayed high tolerance when revealed to high temperatures. While the contrasting one was very susceptible to heat stress. Furthermore, various physiological indicators of photosynthetic systems, like morphological characteristics, chlorophyll contents, antioxidant enzymes, plant endogenous hormones, and proline accumulation, showed significant differences^42,43^. In these lines, the mode of action of heat tolerance in these genotypes will be examined. When plants are exposed to high temperatures, susceptible plants show cellular metabolic imbalances, guiding to a damaged photosynthetic procedure and thus the accumulation of destructive substances within the roots, stems, leaves, and flowers^9^. This accumulation subsequently stunts plant growth and development. However, restoring the traditional growth environment didn't actually induce damage repair while further compromising the plant growth or inducing plant death. Plants that survived the strain displayed stronger vitality, insusceptible cellular homeostasis, normal photosynthesis, and resilience^44,45^. A combination of the differences in endogenous hormone content and gene expression indicated that cucumber genotypes varied in heat tolerance according to various gene expression approaches in reaction to heat stress, which applied a little number of transcription factor families^9,19,20^. Additional studies also demonstrated that ABA may be a typical stress hormone^46^. BR reduces stress damage caused by high-temperature exposure by enhancing pollen fertility, thus, improving crop yield^47^. BR regulates the plant xylem differentiation and architecture in response to heat stress^48^. It also plays a vital role in encouraging fast recovery after exposure to heat stress and decreased oxidative stress^48,49^. Catalase (CAT), SOD (SOD), and peroxidase (POD) are antioxidant enzymes that cover plants from heat-induced oxidative stress. Antioxidant enzymes function as a protection system against deleterious free radicals in plant cells^49,50^. The present study shows that the activities of POD, CAT, and SOD clearly increased when plants were exposed to heat stress, which was consistent with the findings reported^9^. The results demonstrated that the enzyme activities of CAT, POD, and SOD were greatly developed under heat stress, which implies that antioxidant activity enhancement in stressed tissues resulted in low levels of activated oxygen species, which might mitigate injury^15^. Interestingly, heat stress conditions apparently increased the total chlorophyll contents of tolerant genotypes. It confirmed that AOX could remove excessive ROS prompted by heat stress to cut back ROS injury to the membranes. It had been confirmed that antioxidant enzymes in plants work together, and one protective enzyme failed to maintain the balance of active oxygen metabolism in cells^51^. Further plant reactions, such as osmotic regulation, protein stabilization, and hydroxyl radical scavenging were attributed to proline production when plants were subjected to abiotic stresses^43^. The accumulation of proline under heat stress was monitored and associated with stress tolerance, with the content of proline displayed to be naturally higher in stress-tolerant plants than in stress-sensitive ones^43^. In the present investigation, increased levels of proline were marked higher in heat tolerant (HT) genotypes than the heat sensitive (HS) in the heat-stressed cucumber leaves, which suggests the function of proline in tolerance heat stress plants. Dai et al.^52^ found that increased proline levels in heat-stressed leaves enhanced the heat tolerance of cucumber. The plants may have different mechanisms to adapt to heat stress, increasing plant hormones, and antioxidant enzymes in stressed tissues resulting in low levels of activated oxygen species to alleviate injury and accumulation of osmolytes such as proline to stabilize the structure of macro-molecule, decrease the cellular acidification, and elevate resistance ability. At the level of the molecular response, the study revealed a significant increase in the CsGy4G010750 gene with function in superoxide dismutase [Cu-Zn] which agreed with Amin et al.^53^ who revealed a significant increase in gene expression of Cu-Zn SOD and CAT encoding SOD and CAT in cucumber leaves under the heat stress. The results proved that dof zinc finger protein DOF5.7 has a direct effect on heat tolerance in cucumbers. The previous study demonstrated that Dof zinc finger protein family showed documented role in the regulatory networks of plant defense, including responses to diverse biotic and abiotic stresses^20^. Transcription aspects and plant hormones corporately modulate the stress reaction. Moreover, these gene families control the response of the many extremely different metabolic pathways to heat stress in genotypes. Therefore, BRs act as a positive controller under stressful environmental conditions^48^. Endogenous hormone pathways in response to heat under high-temperature stress, genes answerable for protein modification, DNA repair, macromolecule metabolism, and other functions in vivo are specifically up-regulated in thermo-tolerant cucumbers. This is often the response of plants to self-protection and adaptation under external stress^44,48^. Plant hormones play a fancy role in plant stress responses. During this study, hormones and signal transduction pathways were significantly activated following high-temperature exposure. In conclusion, this study exhibited that heat tolerance in cucumbers could be one of the goals to overwhelm the injury of global warming on plant production.

## Conclusions

In light of the challenge of climatic changes and extreme global warming, it has become indispensable for plant breeders to develop outstanding efforts to provide high-tolerant cultivars and hybrids. The mandatory biochemical, physiological, and genetic studies were reported with the heat-tolerant genotypes (P_1_ and P_1_×P_2_) compared with barracuda F_1_ (heat sensitive). This investigation documented that twelve heat tolerance candidate genes in cucumber were predicted in cucumber at the three growth stages 14, 30, and 45 days after transplanting. These genes are closely associated with the synthesis of antioxidant enzymes, (POD, CAT, and SOD) and plant hormones, (GA3, BR, and ABA) which are shown to be produced under heat stress conditions. Overwhelming evidence supports the actual fact that plant hormones recreate important roles in plant biochemical, physiological, and molecular responses to high temperatures. Considering the elevated environmental temperature following global temperature change that threatens plant growth, crop yield, and food productivity worldwide, there's a pressing has to thoroughly examine the plant response to heat stress. Despite the urgent have to improve crop heat tolerance, a really limited number of heat-tolerant varieties are developed. Finally, to attain success, the combined efforts of plant physiologists, molecular biologists, and crop breeders are required.

### Supplementary Information

Supplementary Information.

## Data Availability

The datasets generated during the current study are available in the [NCBI SRA BioProject] repository, accession no [PRJNA380322, PRJNA307098] http://www.ncbi.nlm.nih.gov/Genbank, http://cucurbitgenomics.org/organism/16.
