# Supplementary material for: Morphological, physio-biochemical, and molecular indications of heat stress tolerance in cucumber
Source: Sci Rep. 2023 Oct 31;13:18729. doi: 10.1038/s41598-023-45163-7 (PMC10618462; doi:10.1038/s41598-023-45163-7)
Supplement: Supplementary file 1 — Supplementary Information. [file 41598_2023_45163_MOESM1_ESM.docx]

Supplementary material

**Table S1.** Primers used for RT-qPCR

| ID | **Forward Primer** | **Reverse Primer** |
| --- | --- | --- |
| Actin-F | GTACGACAACGGGCCTTAAA | ATGGGCTTGACAGGTTGTTC |
| *CsGy7G007120* | CTAATGTGTTGAGCGGCAAA | GTCTTACGAACCGCTCTTGC |
| *CsGy2G018910* | CGGCATTACTAAAGCGGAAG | TAGTCCAGAAGGGGGTGTTG |
| *CsGy6G004930* | TTGGATGCCTACACGATCAA | GTATTGGCCAGCTTCACCAT |
| *CsGy6G029150* | CATTGGGAAGGAAGGACTCA | CAATGTCAAGCCCTTGGTTT |
| *CsGy7G015120* | GCTCTTTCAGGAAGCCACAC | GGTAGGGCTCACAAAGTCCA |
| *CsGy7G019290* | TGGCTGTGATGCTTCTATGC | CTGCACAAGAGACGATTCCA |
| *CsGy4G025240* | TTTCTAAGGTCCACCGCAAC | GGGAATTGGGTTTGAAGGAT |
| *CsGy4G025230* | CAAGAAGACGATGGAGCACA | TCCATCAGCATTGGCAATTA |
| *CsGy6G015230* | CCATCCAGTTCGTCCAAGAT | CCTCCATGGTCCTTCTTCAA |
| *CsGy4G005180* | GGGTTACTTCGGACCAGACA | GAAACCCGTCAGCCTATCAA |
| *CsGy1G026400* | ATGACCAAAAACGTCGGAAG | ACTCGCTCGACTCGTTCATT |
| *CsGy4G010750* | AACTTCTGCTTCGACCTCCA | AACTCGTGCGAGAAATGCTT |
| *CsGy7G007120* | AAAGGACGGACAACCAAGTG | GCCAGCTACGTCTCCAAGTC |

**Table S2.** Genes linked to temperature stress having high homology with other genes selected from different plant species.

| **Gene ID** | **High homology** |
| --- | --- |
| CsGy2G003240 | *Cucumis sativus, Cucumis melo* |
| CsGy2G025870 | *Cucumis sativus, Cucumis melo, Cucurbita maxima, Cucurbita pepo* subsp*. pepo, Momordica charantia,, Cucurbita moschata* |
| CsGy2G025820 | *Cucumis sativus, Cucumis melo* |
| CsGy4G005180 | *Cucumis sativus, Cucumis melo Momordica charantia Cucurbita pepo* subsp*. pepo* |
| CsGy4G025230 | *Cucumis sativus, Cucumis melo Cucurbita moschata* |
| CsGy4G010750 | *Cucurbita maxima, Cucurbita pepo subsp.* pe*po, Luffa aegyptiaca, Cucumis melo*, *Corchorus capsularis,_Arabidopsis thaliana* |
| CsGy6G029150 | *Cucumis sativus,* MSBR*_ Momordica charantia,* and CMBR*_ Cucurbita moschata* |
| CsGy4G022650 | *Cucumis sativus, Cucumis melo, Momordica charantia, Cucurbita maxima, Cucurbita pepo* subsp*. pepo,* |
| CsGy6G022520 | *Cucumis sativus, Cucumis melo, Cucurbita maxima, Cucurbita moschata, Cucurbita pepo* subsp*. pepo* |


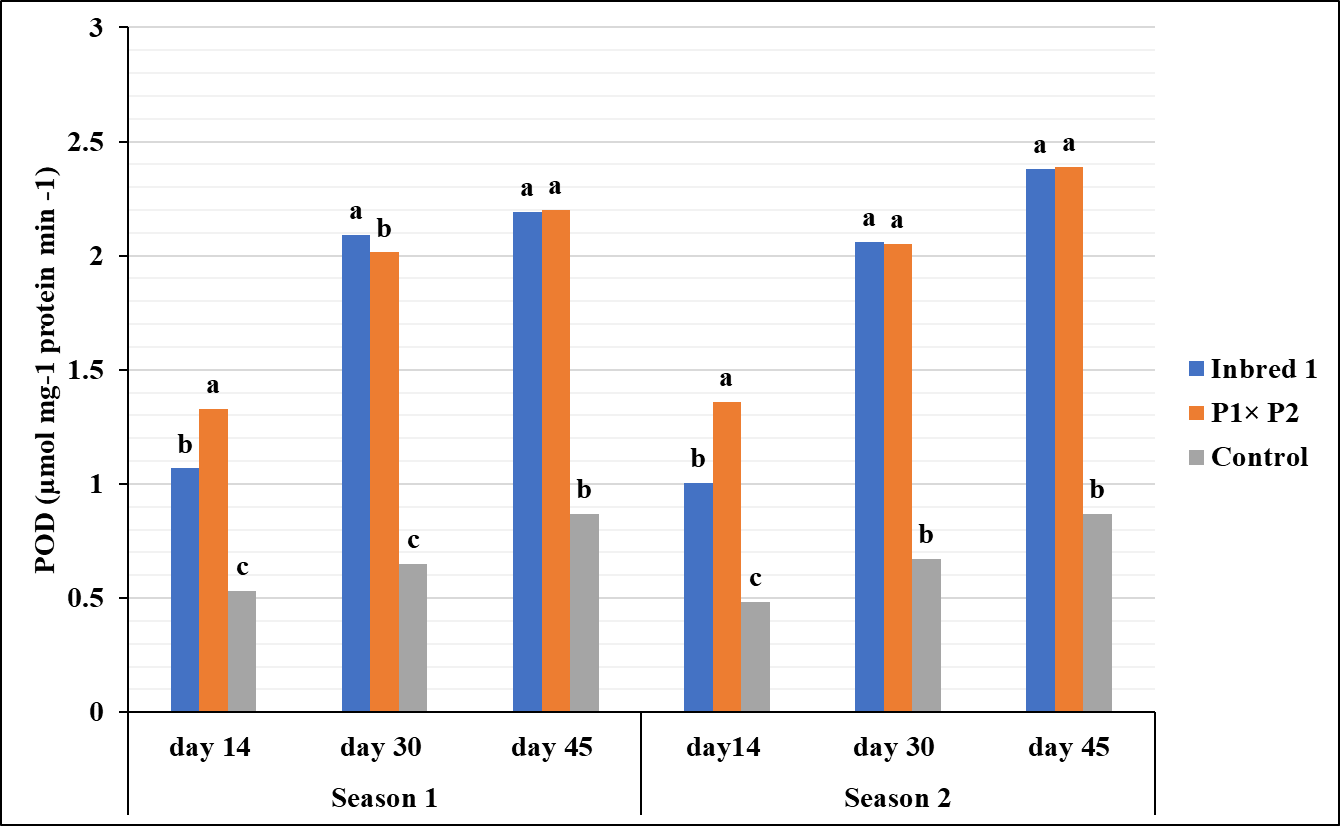


**Figure S1:** Changes in POD (peroxidase) enzyme activities in of parent P_1_, hybrid P_1_×P_2_ and control cucumber leaves under normal condition (non-heat stress condition) at 14, 30, and 45 days. Means (± SE) followed by the same letter refer to insignificant differences at *p* ≤0.05 (LSD test) of two season.


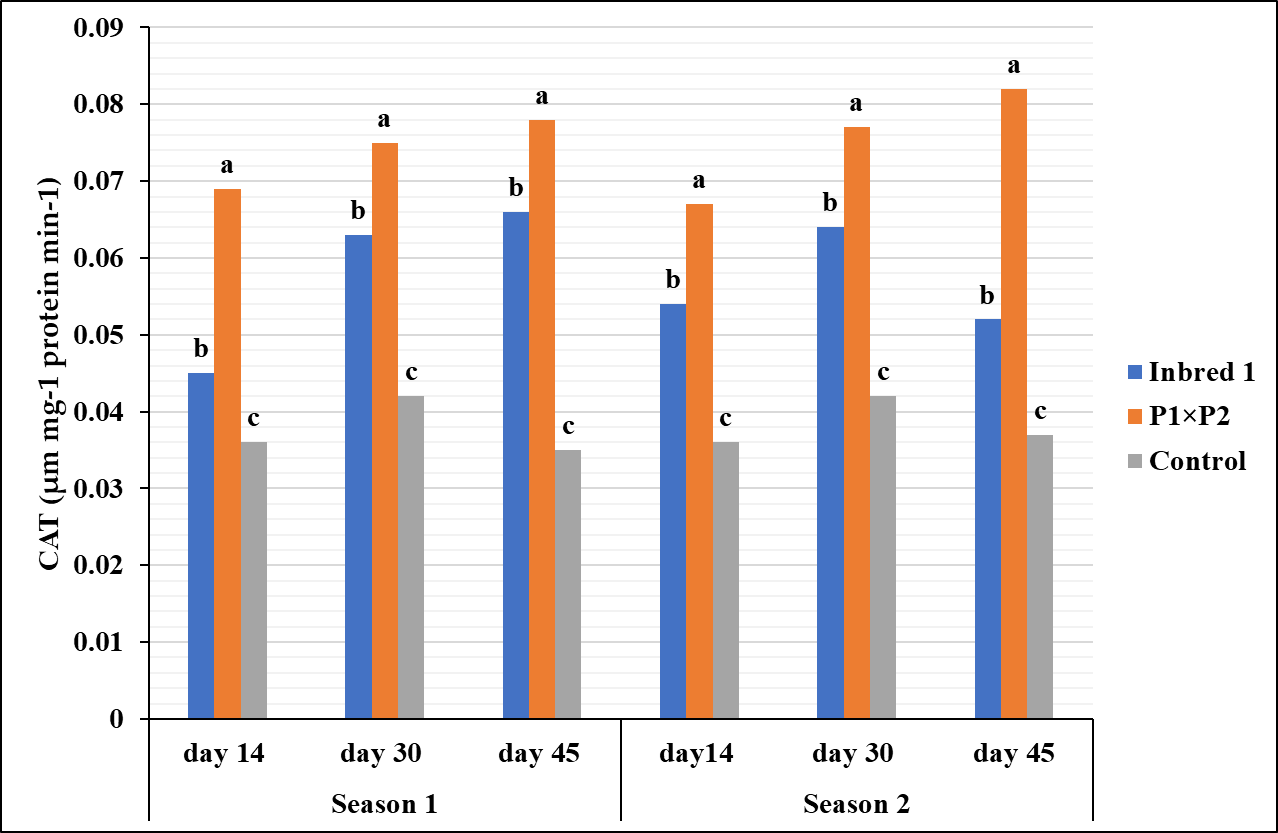


**Figure S2:** Changes in CAT (catalase), enzyme activities in of parent P_1_, hybrid P_1_×P_2_ and control cucumber leaves under normal condition (non-heat stress condition) at 14, 30, and 45 days. Means (± SE) followed by the same letter refer to insignificant differences at *p* ≤0.05 (LSD test) of two season.


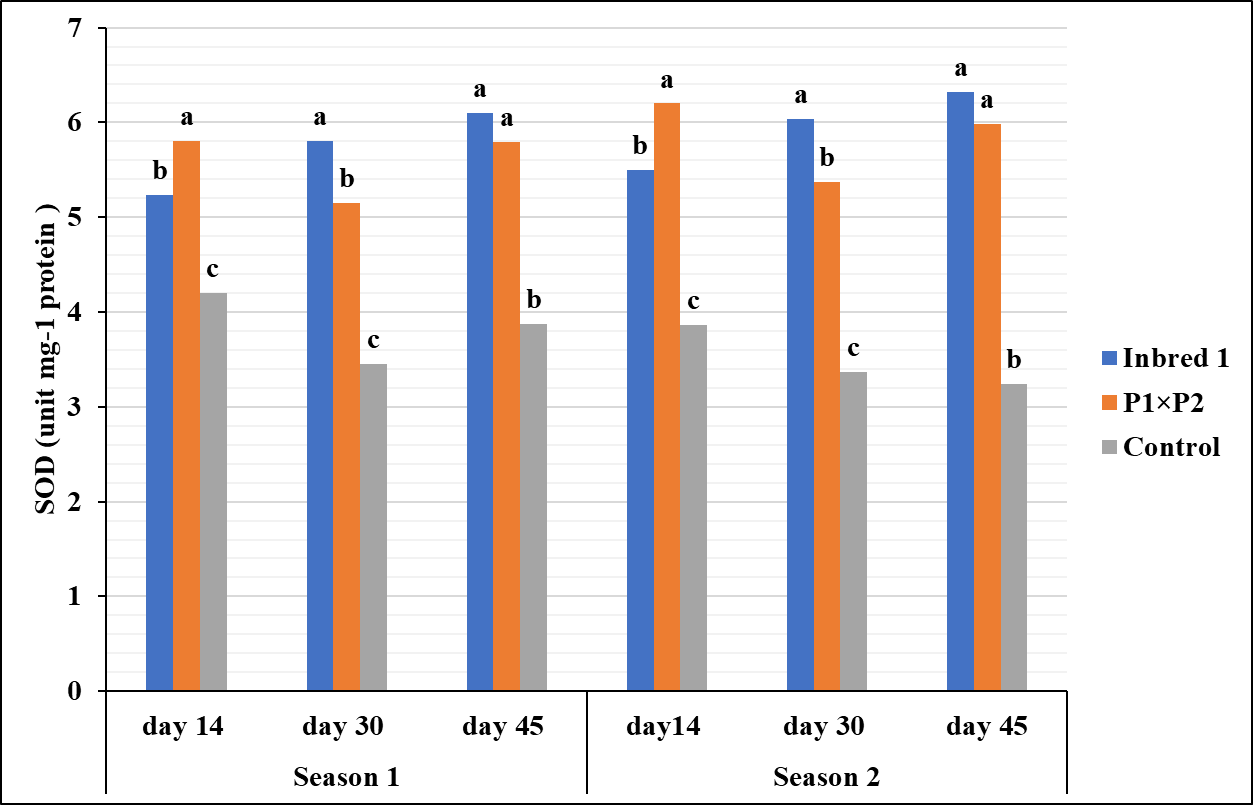


**Figure S3****:** Changes in SOD (superoxide dismutase) enzyme activities in of parent P_1_, hybrid P_1_×P_2_ and control cucumber leaves under normal condition (non-heat stress condition) at 14, 30, and 45 days. Means (± SE) followed by the same letter refer to insignificant differences at *p* ≤0.05 (LSD test) of two season.
